# Book Notices and Reviews

**Published:** 1865-04

**Authors:** 


### BOOK NOTICES AND REVIEWS.

*Therapeutics and Materia Medica:* A Systematic Treatise on the Action and Uses of Medicinal Agents, including their Description and History. By ALFRED STILLE, M. D. Second Edition. Revised and Enlarged. In Two Volumes. From the Publishers, Blanchard & Lea, Philadelphia: through Wm. B. Keen & Co., Chicago.

The appearance of a new edition of Prof. Stille's work offers us an opportunity to commend it to the profession—though such commendation may seem superfluous—for since the appearance of the first edition, it has been so often and ably reviewed, and always with favor; it enriches the libraries of so many physicians and students, and is so familiar to all, that we might be justified in passing the advent of a new edition over in silence. The second edition differs from the first in this, that the several articles which go to make up the treatise have been carefully revised, and while some unimportant things have been omitted, above one hundred pages of new matter have been added to the work, and the nomenclature and formulæ have been made to conform to the new edition of the Pharmacopœia. To us, the Author seems to have exhibited good judgment in devoting his attention largely to the *therapeutics* of medicines, which he has greatly illustrated by copious citations of their physiological action, leaving the minute details of the Materia Medica to be gleaned from more elementary works. What physicians in practice desire to obtain from works of this nature is, what medicines shall they select and how shall they use them for the cure of disease, and this, Stille teaches, and teaches it well.

The work is evidently prepared with great labor and care, and the Author seems eminently qualified for his task, not alone by his superior attainments, and the facility and elegance with which he expresses his ideas, but in that he appears to be uninfluenced by prejudice, and has no pet theories to cherish or defend, and withal his mind is imbued with a salutary skepticism, which prevents him from accepting an opinion simply because it has been long entertained, or that is commended alone by its novelty. There is, perhaps, no department of medical science in which the American intellect has shown more conspicuously than in this, and the recent labors of such men as Stille and Wood, in therapeutical investigations, have emancipated us, in this particular, from our dependence on foreign pens, and there is now no such treatise on Materia Medica, that is much sold or read in this country, and to be released from all external dependence,but especially in what pertains to the nobler achievements of the intellect, is a fit subject of rejoicing to every American. Again we would recommend the work to the *study* of those whose vocation it is to treat disease.

*A Dictionary of Medical Science:* Containing a concise Explanation of the various subjects and terms of Anatomy, Physiology, Hygiene, Therapeutics, Pharmacology, Pharmacy, Surgery, Obstetrics, Medical Jurisprudence and Dentistry. Notices of Climate, and of Mineral Waters, Formulæ for Officinal, Empirical and Diatetic preparations, with the Accentuation and Etymology of the terms, and the French and other synonyms, so as to constitute a French as well as English Medical Lexicon. By Robley Dunglison, M. D., LL.D., Professor of the Institutes of Medicine, etc., in the Jefferson Medical College, of Philadelphia. Thoroughly revised and very greatly modified and augmented. Philade.phia: Blanchard & Lea. 1865.

The pages of the *Journal* have frequently contained notices of this work, and always commendatory, as the successive editions have appeared. The value of the present edition has been greatly enhanced by the introduction of new subjects and terms, and a more complete etymology and accentuation, which renders the work not only satisfactory and desirable, but indispensable, to the Physician.

For sale by W. B. Keen & Co., 146 Lake street.

---

*The New York Medical Journal.*—We hail with pleasure the appearance of a Medical Journal in the *City of New York*, a place of considerable importance, on account of the number of its inhabitants, its wealth and commercial advantages, but for some time without a Medical periodical of its own. The fact that a *Medical Journal* has now been started there we consider as praiseworthy and honorable to the enterprise of our brethren in that distant city. We hope that this experiment may succeed. But seriously, judging from the mechanical execution and contents of the initial number, which is before us, it is to be in every particular worthy the Profession and the city whence it issues. "The New York Medical Journal" will be published on the first of every month, and will contain not less than 80 octavo pages in each number. Terms, five dollars a year. Address the "Editor, N. Y. Medical Journal." We wish the "Journal" all success, which is ensured by the long list of contributors, including the names of many of the ablest men in our ranks.

